# Ecdysteroids Regulate the Levels of Molt-Inhibiting Hormone (*MIH*) Expression in the Blue Crab, *Callinectes sapidus*

**DOI:** 10.1371/journal.pone.0117278

**Published:** 2015-04-07

**Authors:** Sirinart Techa, J. Sook Chung

**Affiliations:** Institute of Marine and Environmental Technology, University of Maryland Center for Environmental Science, 701 E. Pratt Street, Columbus Center, Baltimore, Maryland, 21202, United States of America; Tel Aviv University, ISRAEL

## Abstract

Arthropod molt is coordinated through the interplay between ecdysteroids and neuropeptide hormones. In crustaceans, changes in the activity of Y-organs during the molt cycle have been regulated by molt-inhibiting hormone (MIH) and crustacean hyperglycemic hormone (CHH). Little has been known of the mode of direct effects of ecdysteroids on the levels of MIH and CHH in the eyestalk ganglia during the molt cycle. This study focused on a putative feedback of ecdysteroids on the expression levels of *MIH* transcripts using *in vitro* incubation study with ecdysteroids and *in vivo* RNAi in the blue crab, *Callinectes sapidus*. Our results show a specific expression of *ecdysone receptor* (*EcR*) in which *EcR1* is the major isoform in eyestalk ganglia. The initial elevation of *MIH* expression at the early premolt stages is replicated by *in vitro* incubations of eyestalk ganglia with ecdysteroids that mimic the intrinsic conditions of D_0_ stage: the concentration (75 ng/ml) and composition (ponasterone A and 20-hydroxyecdysone at a 3:1 (w:w) ratio). Additionally, multiple injections of *EcR1-dsRNA* reduce *MIH* expression by 67%, compared to the controls. Our data provide evidence on a putative feedback mechanism of hormonal regulation during molting cycle, specifically how the molt cycle is repeated during the life cycle of crustaceans. The elevated concentrations of ecdysteroids at early premolt stage may act positively on the levels of *MIH* expression in the eyestalk ganglia. Subsequently, the increased MIH titers in the hemolymph at postmolt would inhibit the synthesis and release of ecdysteroids by Y-organs, resulting in re-setting the subsequent molt cycle.

## Introduction

Endocrine systems normally have feedback controls to regulate their balance in the organisms. In vertebrates, steroid hormones such as estrogens, glucocorticoids, and androgens regulate their production through negative feedback on neuroendocrine axes [1]. In insects, prothoracicotropic hormone (PTTH) that is produced from the brain and released by corpora cardiaca stimulates the prothoracic gland for ecdysteroidogenesis. Ecdysteroids in turn positively regulate PTTH levels in *Manduca sexta* [2–5]. As a short-loop feedback, ecdysteroids also act on the prothoracic gland in a concentration-dependent manner: lower levels for stimulation and higher for inhibition in *M*. *sexta* and *Pieris brassicae* [1,6–8].

Life stages of arthropods continue through the recapitulated molting process. Molting is is hormonally regulated and involves cell division, synthesis and deposition of new cuticle after shedding of the old one [9–11]. Two members of the crustacean hyperglycemic hormone (CHH) family that originate from the endocrine tissue, the X-organ sinus gland system located within the eyestalk, are involved in the regulation of molting: 1) CHH and 2) molt-inhibiting hormone (MIH) [11–15]. MIH and CHH suppress the synthesis and release of ecdysteroids by Y-organs [16,17]. The hemolymph concentrations of CHH and MIH show a close association with the levels of ecdysteroids during the molt cycle in the European green crab, *Carcinus maenas* [18]. However, the regulatory mechanism underlying *MIH* expression and MIH secretion is still unknown in crustaceans.

Ecdysteroids, arthropods’ molting hormones, are secreted by crustacean Y-organs that are homologous of insect prothoracic glands. The levels of hemolymphatic ecdysteroids are positively related to molt stages in many decapod crustaceans including *Cancer magister*, *C*. *maenas*, *Callinectes sapidus*, *Daphnia magna*, *Hyas araneus*, *Homarus americanus*, *Metopograpsus messor*, and *Procambarus clarkii* [19–26]. Y-organs secrete inactive forms of ecdysteroids: ecdysone, and 25-deoxyecdysone (25-dE) [27–30] that are subsequently hydroxylated in the peripheral tissues to active forms: 20-hydroxyecdysone (20-HE) and ponasterone A (PoA), respectively [20,31–33]. 20-HE is known to be the main active ecdysteroid in insects. However, the hemolymph of a given crustacean species carries more than one active form. In the premolt hemolymph of *C*. *sapidus* and *C*. *maenas*, PoA is measured as the major type of ecdysteroid and then 20-HE as the second form [26,31,34].

Ecdysteroids have been known to affect the activity of Y-organs as well as that of the eyestalk ganglia in crustaceans [35,36]. 20-HE influences the Y-organ activity in *C*. *maenas*, *Orconectes limosus* and *Uca pugilator*, positively or negatively depending on its concentrations [37–39]. A putative positive feedback of ecdysteroids on eyestalk neuropeptides is suggested in *Cancer antennarius* and *U*. *pugilator* [35,36]. Interestingly, at premolt stages, the concentrations of ecdysteroids as well as the ratio between the two active forms are changed. At the mid-premolt (D_2_) stage of *C*. *sapidus*, amounts of PoA to 20-HE are observed at a 3:1 ratio [34]. To date, it has not yet been studied in the regulation of the molt control of decapod crustaceans, specifically how such inherent changes in ecdysteroid titers and the ratio between PoA and 20-HE could affect the transcription and translation of MIH/CHH in the eyestalk ganglia during the molt cycle.

The presence of two active ecdysteroids in *C*. *sapidus* together with multiple forms of *EcR/RXR* and their binding to ligand indicate the involvement of this hormone in various physiological processes in this species. The presence of putative multiple isoforms of *EcR* and *RXR* seems common as it is found in several decapod crustaceans [40–45]. In *C*. *sapidus*, *CasEcR1* differs from *CasEcR2* by the property of the putative ligand binding pockets (LBP) in that the LBP of the former contains more hydrophilic amino acids (aa) than that of the latter. *CasRXR* isoforms are characterized by insertion in either DBD (5 aa) or LBD (45 aa) or both, resulting in four different isoforms. Additionally, most of the internal tissues of this species express multiple isoforms of *EcRs* and *RXRs* [44,46].

EcR is known to bind directly to ecdysteroids, whereas RXR facilitates the liganded EcR binding on its responsive element, AGGTCA motif of DNAs [47,48]. Binding of RXR to a ligand(s) seems to be unclear. It is suggested that *Carcinus* RXR may bind directly to methyl farnesoate (MF) [49], while *Daphnia* RXR does not [50]. Interestingly, levels of *EcR* itself and USP, a homolog of RXR in *D*. *melanogaster* [51,52] are regulated by ecdysteroids. In crustaceans, upregulation of *EcR* was reported in *U*. *pugilator* limb bud after being incubated in ecdysteroids [53]. To date, it has not yet been examined if the elevated levels of total ecdysteroids or a specific type of ecdysteroid in the hemolymph influence the levels of *EcR* expression in relation to the molt regulation of decapod crustaceans.

In this study, we aimed to better understand the hormonal regulation of molting, the process which recapitulates throughout the life cycle of crustaceans using the hatchery-raised blue crab, *C*. *sapidus* with tractable life and molt stage as a model animal. Specifically, to define a putative feedback mechanism, we focused on the interplay between two endocrine systems involving CasMIH from eyestalk ganglia and ecdysteroids from Y-organs. We first examined to test if ecdysteroids influence the expression of *CasMIH* transcripts in eyestalk ganglia using an *in vitro* incubation. We then also determined the effect of multiple injections of *CasEcR-dsRNA* on *CasMIH* expression in the eyestalk ganglia.

## Results

### Levels of molt-inhibiting hormone (*CasMIH*) expression in eyestalk ganglia and of CasMIH in hemolymph during the molt cycle

**Fig 1:**
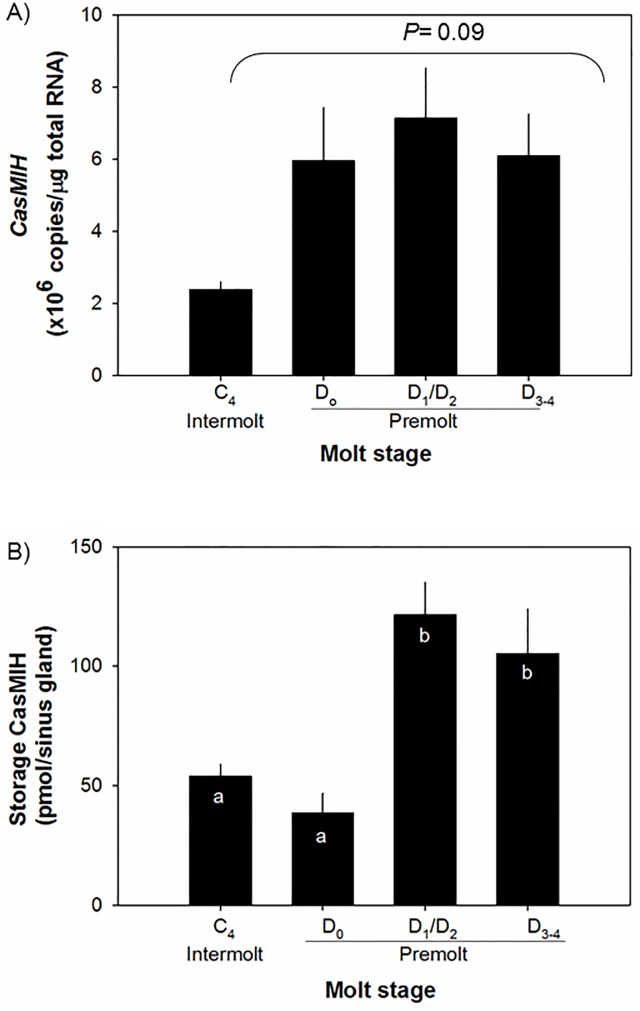
Levels of *CasMIH* in eyestalk ganglia (A, n = 7–8) and in the sinus gland (B, n = 7–21) during juvenile molt by qRT-PCR assay and ELISA, respectively. Each cDNA sample containing 25 ng total RNA equivalent was assayed in duplicate. The expression levels are represented as copies/μg total RNA. The data are presented as mean ± SE. Native CasMIH for ELISA standards was prepared as described [75]. All data were subjected to a normality test using the Shapiro-Wilk test (SigmaPlot). Statistical significance was accepted at *P* < 0.05 and noted with letters.

Levels of *CasMIH* transcripts tend to increase during the molt cycle with a *P*-value = 0.09 (Fig 1A). At intermolt (C_4_), the lowest levels of *CasMIH* are measured at 2.4 ± 0.2 x 10^6^ copies/μg total RNA (n = 8). These levels are increased to 6.0 ± 1.5 x10^6^ copies/μg total RNA (n = 8) at D_0_ stage (*P* = 0.09) and reach to 7.1 ± 1.4 x10^6^ copies/μg total RNA (n = 22) at D_1_/D_2_ stage and decline to 6.1 ± 1.2 x10^6^ copies/μg total RNA (n = 7) at D_3-4_ stages.

The amounts of CasMIH protein stored in the sinus gland during the molt cycle are presented in Fig 1B. At the intermolt and early premolt (D_0_) stages, CasMIH levels are similar and range from 53.9 ± 5.0 to 38.4 ± 8.3 pmol/sinus gland (n = 10–12), respectively. At the mid-premolt stage (D_1_/D_2_), CasMIH levels increase significantly to 121.5 ± 13.6 pmol/sinus gland (n = 21) and remain high at 105.3 ± 18.5 pmol/sinus gland (n = 7) during the late premolt (D_3/4_) stage.

**Fig 2:**
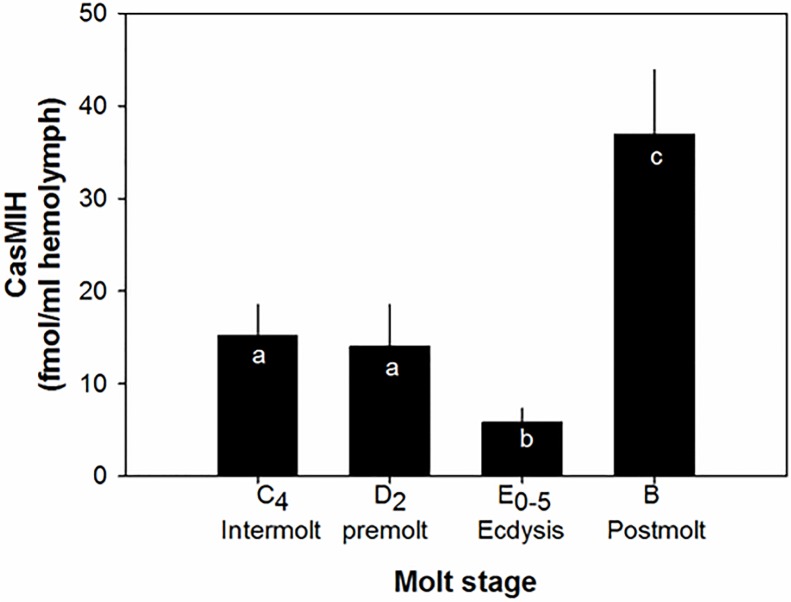
Hemolymph titers of CasMIH during the molt cycle (n = 6–14). The data are presented as mean ± SE. Native CasMIH for RIA standards was prepared as described [75]. All data were subjected to a normality test using the Shapiro-Wilk test (SigmaPlot). Statistical significance was accepted at *P* < 0.05 and noted with letters.

The concentrations of CasMIH in hemolymphs also change during the molt cycle. CasMIH titers are similar during intermolt (n = 8) and mid-premolt D_2_ stages (n = 14), ranging from 14.1 to 15.2 pM (Fig 2). At early ecdysis (E_0-5%_), the CasMIH titers are significantly reduced to 5.8 ± 1.5 pM (n = 11), compared to those measured at intermolt and mid-premolt stages. At post molt (B) stage, the levels of CasMIH are significantly elevated to 36.7 ± 7.0 pM (n = 6).

### Endogenous changes in ecdysteroid concentrations in eyestalk ganglia and Y-organs at different molt stages

**Fig 3:**
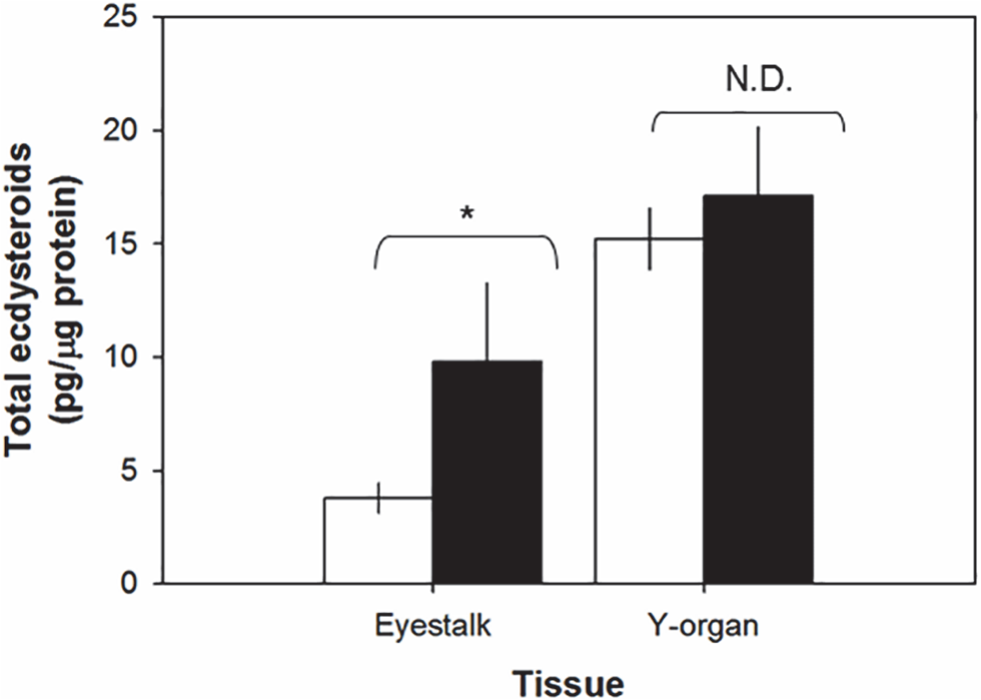
Levels of ecdysteroid presence in the tissues assayed by Ecd-RIA. Eyestalk and Y-organs from intermolt (white bar, n = 4–6) and premolt (black bar, n = 6) stages of juvenile *C*. *sapidus*. Protein contents in eyestalk and Y-organs were estimated using colorimetric assay, modified Lowry method (Pierce 660). All data were subjected to a normal distribution using the Shapiro-Wilk test (SigmaPlot). Statistical significance was accepted at *P* < 0.05 and noted with an asterisk. N.D. = not different.

The ecdysteroid contents vary by tissues and molt stages (Fig 3). Levels of ecdysteroids in eyestalk ganglia and Y-organ at intermolt stage do not differ statistically: 2.3 ± 1.0 pg/μg eyestalk ganglia proteins (n = 4) and 9.8 ± 3.4 pg/μg Y-organ proteins (n = 6). Ecdysteroid levels increase significantly (*P* = 0.05) by three folds to 9.8 ± 3.5 pg/μg eyestalk ganglia proteins (n = 6) at early and mid-premolt (D_0_/D_1_) stages, compared to those estimated at intermolt. However, the ecdysteroid levels in Y-organs are increased only to 17.1 ± 3.0 pg/μg Y-organ proteins at early and mid-premolt (D_0_/D_1_) stages. These values do not differ significantly, compared to those determined at intermolt. The hepatopancreas, as a reference tissue, has similar levels of ecdysteroids at intermolt: 38.1 ± 5.7 pg/ μg hepatopancreas proteins (n = 5) and early and mid-premolt (D_0_/D_1_) stages: 36.6 ± 4.3 pg/ μg hepatopancreas proteins (n = 7).

### Effects of ecdysteroids on levels of *CasMIH* expression

**Fig 4:**
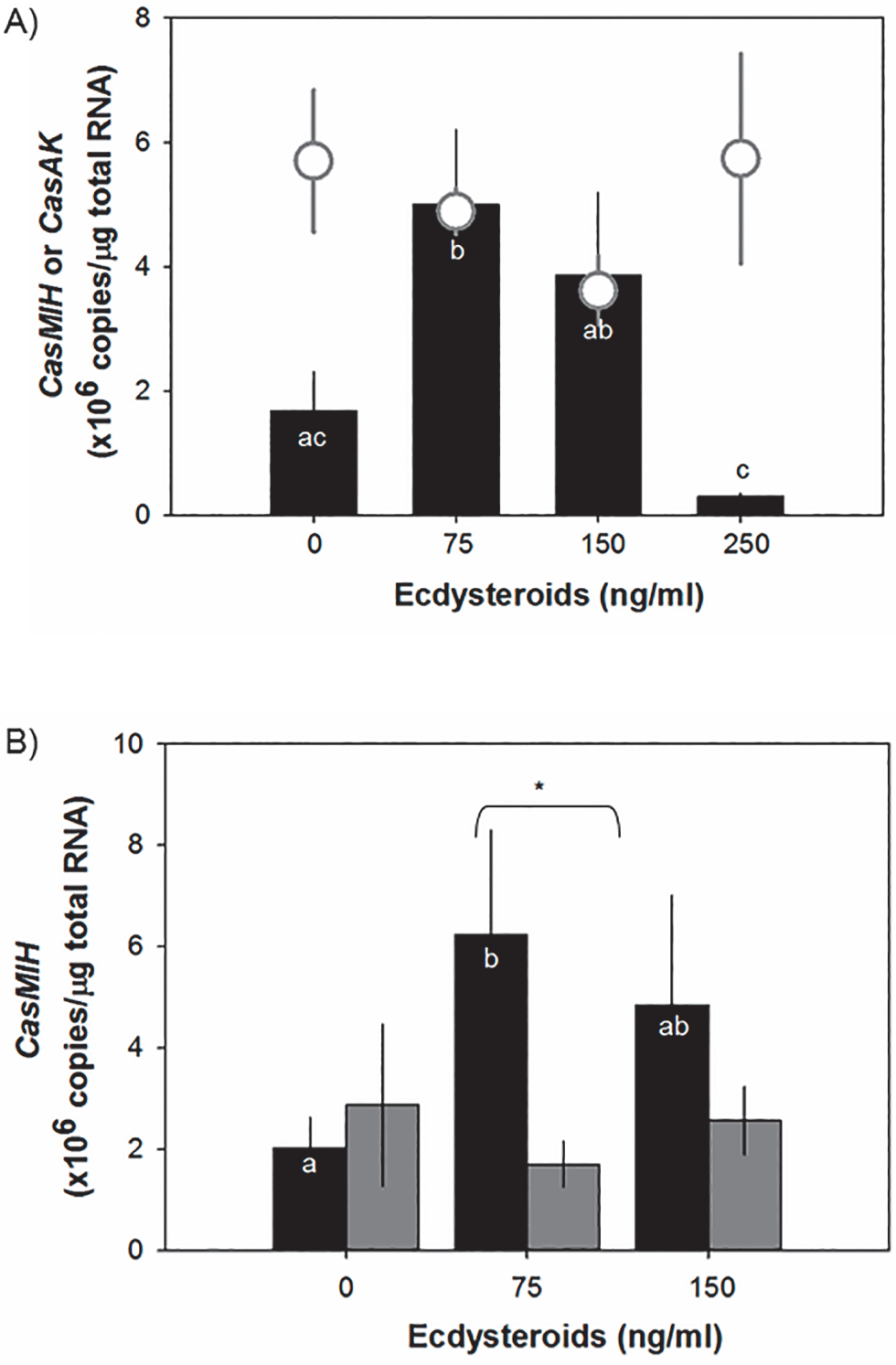
Transcriptional levels of *CasMIH* in eyestalk ganglia after incubation with different concentrations (A) and ratios (B) of active ecdysteroids by qRT-PCR assay. A) *CasMIH* levels (black bar, n = 5–8) in eyestalk ganglia that were incubated with different ecdysteroid concentrations composed of a 3:1 ratio (w:w) of PoA: 20-HE. The ecdysteroid concentrations of 75, 150, and 250 ng/ml mimic the D_o_, D_1_, and D_2_ stages, respectively. *C*. *sapidus arginine kinase* (*CasAK*) expression (open gray circle, n = 6–8) was quantified in the same cDNA samples. B) *CasMIH* levels in eyestalk ganglia incubated with different ratios (w:w) of PoA and 20-HE: 3:1 (black bar, n = 3–9) and 1 to 3 (gray bar, n = 3–9). Each cDNA sample containing 25 ng total RNA equivalent was assayed in duplicate. The expression levels are represented as copies/μg total RNA. The data are presented as mean ± SE. All data were subjected to a normality test using the Shapiro-Wilk test (SigmaPlot). Statistical significance was accepted at *P* < 0.05 and noted with letters and asterisks.

The effect of different applied ecdysteroid levels on *in vitro* incubated intermolt eyestalk ganglia is presented in Fig 4. The ecdysteroids were applied at the composition of PoA:20-HE at a 3:1 ratio, mimicking the concentrations at various molt stages (D_0_, D_1_, and D_2_) (Fig 4A). At 75 ng/ml ecdysteroid concentration (D_0_), levels of *CasMIH* transcript are significantly up-regulated to 5.0 ± 1.2 x10^6^ copies/μg total RNA (n = 7), compared to those of the control (1.7 ± 0.6 x10^6^ copies/μg total RNA, n = 8). At higher concentrations of ecdysteroids at 150 and 250 ng/ml, levels of *CasMIH* transcript tend to decrease to 3.9 ± 1.3 x10^6^ copies/μg total RNA (n = 16) and 0.3 ± 0.1 x10^6^ copies/μg total RNA (n = 5), respectively. However, these reduced *CasMIH* levels do not differ from the controls. Levels of *CasAK* transcript measured in the same sample cDNAs remain constant, ranging from 5.1 ± 0.6 x10^6^ copies/μg total RNA (n = 6) with the controls; 4.5 ± 0.5 x10^6^ copies/μg total RNA (n = 8) at 75 ng/ml; 3.9 ± 0.6 x10^6^ copies/μg total RNA (n = 8) at 150 ng/ml; and 5.7 ± 1.7 x10^6^ copies/μg total RNA (n = 8).

Stimulatory effect of ecdysteroid ratios on *CasMIH* transcription is further determined with PoA and 20-HE at 1:3 (Fig 4B). The ratio of PoA:20-HE is critical to stimulate *CasMIH* transcription, apparently only the 3:1 ratio induces *CasMIH* transcription. At 75 ng/ml, the *CasMIH* levels are increased significantly (*P*<0.05) to 6.2 ± 2.1 x10^6^ copies/μg total RNA (n = 9), compared to the controls (2.1 ± 0.6 x10^6^ copies/μg total RNA, n = 3). In contrast, the ratio of PoA:20-HE at 1:3 (Fig 4B, gray bar) has no effect on the *CasMIH* transcription: 1.7 ± 0.4 x10^6^ copies/μg total RNA (n = 9). Similarly, the incubation with ecdysteroids composed of PoA:20-HE at 3:1 at 150 ng/ml (~ D_1_ stage) slightly elevates *CasMIH* transcription (4.9 ± 2.2 x10^6^ copies/μg total RNA, n = 9) but not at 1:3 (2.6 ± 0.7 x10^6^ copies/μg total RNA, n = 10).

### Temporal distributions of ecdysone receptor (*CasEcR*) isoforms in eyestalk ganglia during the molt cycle

**Fig 5:**
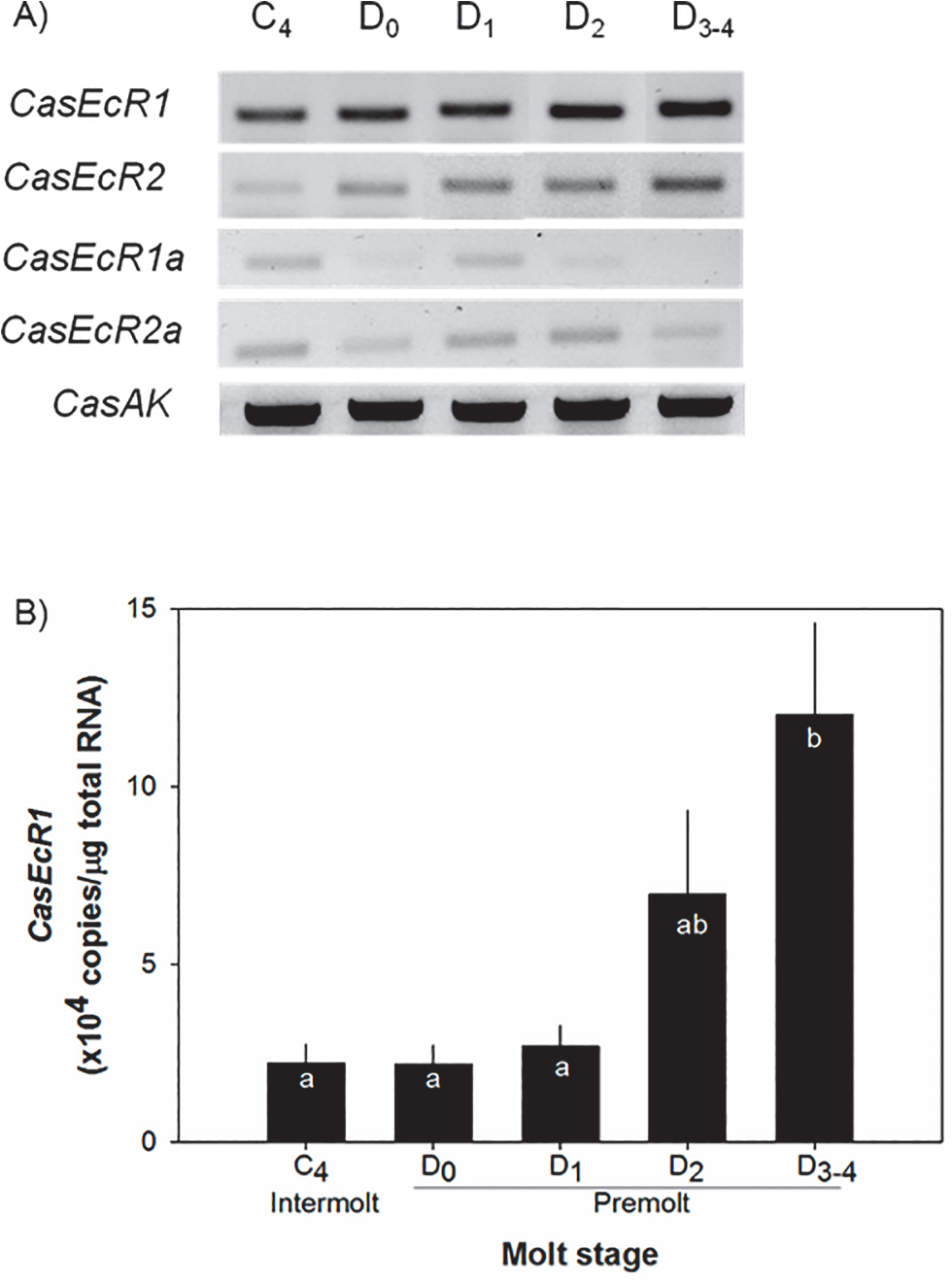
Temporal distributions of four isoforms of *CasEcRs* with *CasAK*, as a reference gene (A) and levels of *CasEcR1* expression in eyestalk ganglia during a juvenile molt using qRT-PCR assay. Specific primers of each isoform are listed in Table 1. Sample cDNAs each containing 25 ng total RNA equivalent were assayed in duplicate. The expression levels are represented as copies/μg total RNA. The data are presented as mean ± SE (n = 6–8). All data were subjected to a normality test using the Shapiro-Wilk test (SigmaPlot). Statistical significance was accepted at *P* < 0.05 and noted with letters.

In eyestalk ganglia, four forms of *CasEcR* expression patterns vary by molt stages. *CasEcR1* serves as the major form, followed by *CasEcR2*. These two expression levels show a gradual incease at premolt stages (D_0_-D_3-4_, Fig 5A and 5B). However, *Ca*s*AK* expression in this tissue is constant throughout the molt cycle.

Levels of *CasEcR1* transcript remain similar at intermolt and mid-premolt (D_2_) ranging from 2.2 to 7.0 x10^4^ copies/μg total RNA (n = 6–8). Then the levels are elevated significantly to 1.2 ± 0.2 x10^5^ copies/μg total RNA (n = 7) at late premolt D_3-4_ stages (*P*<0.01).

### Effects of *CasMIH-* and *CasEcR1-dsRNA* on *CasMIH* expression in the eyestalk ganglia and the molt interval

**Fig 6:**
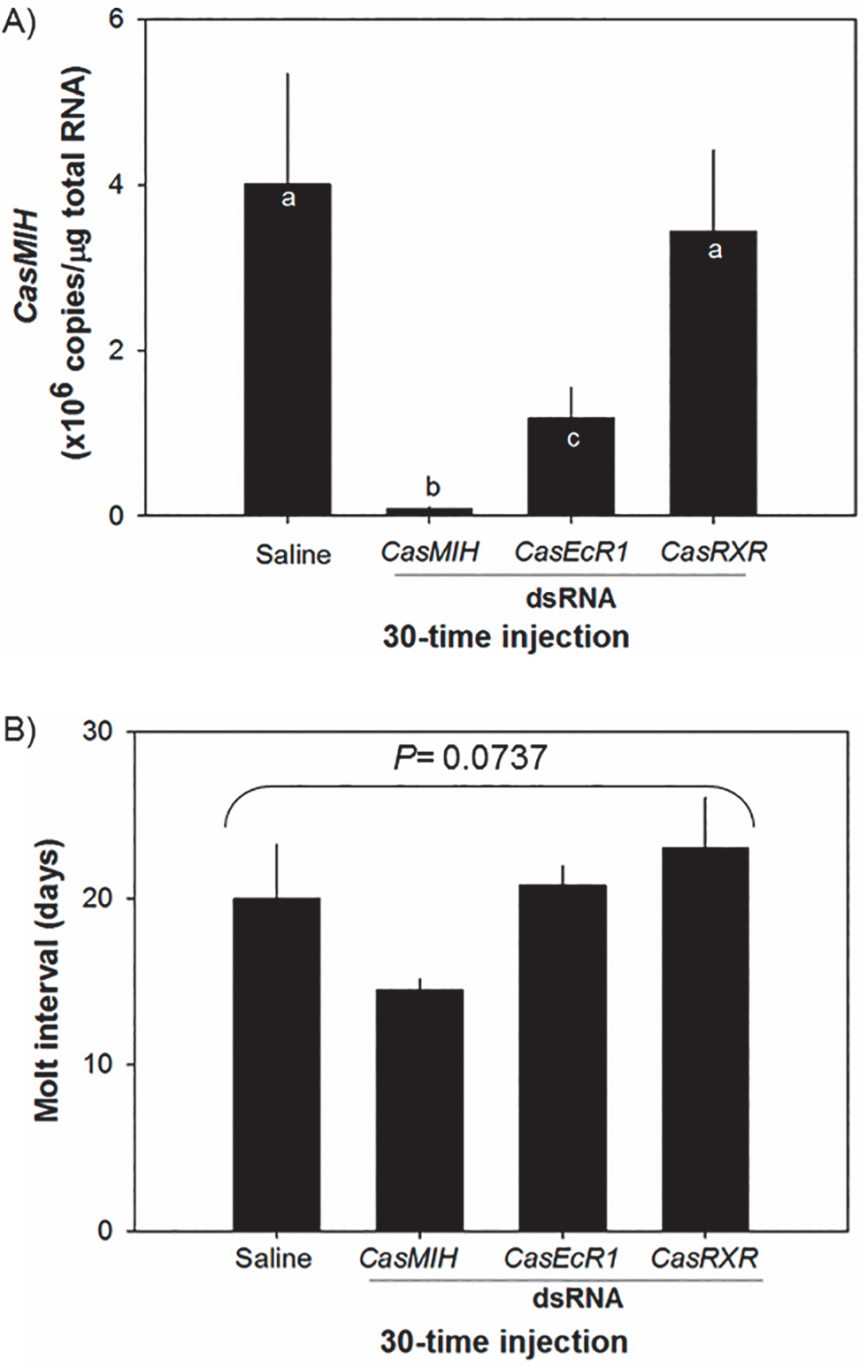
Expression levels of *CasMIH* in the eyestalk ganglia (A) and molt interval (B) after being administered with *dsRNAs* (15 μg each injection, ~30 times over 60 days) by qRT-PCR assay. Each cDNA sample containing 25 ng total RNA equivalent was assayed in duplicate. The expression levels are represented as copies/μg total RNA. The data are presented as mean ± SE (n = 5–9). All data were subjected to a normality test using the Shapiro-Wilk test (SigmaPlot). Statistical significance was accepted at *P* < 0.05 and noted with letters.

A preliminary time-course study with multiple injections of *CasMIH-dsRNA* significantly reduced the levels of *CasMIH* transcripts (S1 Fig) whereas *CasRXR-dsRNA* had no effect on the levels of *CasMIH* transcripts (data not shown). Multilple *CasMIH*-*dsRNA* injections (15 μg per injection, ~30 times over 60 days) reduce *CasMIH* expression by ~50-times: 8.0 ± 3.0 x10^4^ copies/μg total RNA (n = 9), compared to those that received saline (4.0 ± 1.3 x10^6^ copies/μg total RNA, n = 6) and, as a reference, *CasRXR-dsRNA* (3.4 ± 1.0 x10^6^ copies/μg total RNA, n = 5). Interestingly, *CasEcR1-dsRNA* significantly reduces *CasMIH* levels (1.2 ± 0.4 x10^6^ copies/μg total RNA (n = 9), compared to the controls. However, *CasRXR-dsRNA* injections have no effect on the levels of *CasMIH* expression (Fig 6A).

The molt interval of these experimental groups is shown in Fig 6B. The animals injected with *CasMIH-dsRNA* have molt intervals with 14.5 ± 3.0 days (n = 4). These values are not significantly different (*P* = 0.0737) from those of other groups including the control with 20.0 ± 3.2 days (n = 4), *CasEcR1-dsRNA* with 20.8 ± 1.2 days (n = 5), and *CasRXR-dsRNA* with 23.0 ± 3.0 days (n = 5).

### Sequencing analysis of the upstream promoter region of *CasMIH* gene

**Fig 7:**
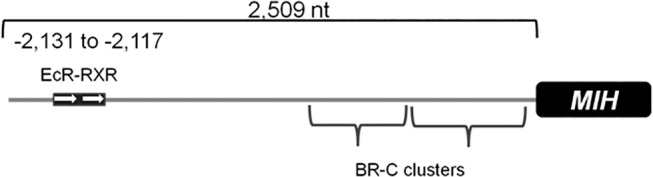
A schematic diagram of upstream promoter region of *CasMIH* gene (GenBank Accession no. KJ813010) containing putative DNA binding sites. The putative binding sites of ecdysteroid-responsive factors are predicted (http://consite.genereg.net/). White arrows represent a direct repeat of AGGTCA motifs for EcR-RXR binding. The binding site of broad complex (BR-C) is located in clusters.

The upstream promoter region of *C*. *sapidus MIH* gene was extended 2,509 nt from the start codon (Fig 7). Binding sites of two transcription factors that respond to ecdysteroids are predicted in this region. Direct repeat of AGGTCA motifs, serving as the binding site of EcR and RXR, is predicted at the region between -2,131 and -2,117 nt (two arrows in Fig 7). The binding sites for broad-complex, an early ecdysteroid responsive gene, are clustered in tandem repeats at the proximal region (-1,567 to -430) and located close to the start codon of *CasMIH*.

## Discussion

Endocrine systems usually involve feedback mechanisms to maintain physiological homeostasis. The present study describes the interplays between the levels of ecdysteroids and *MIH* in the blue crab, *C*. *sapidus*. Our results demonstrate that *CasEcRs* are elevated in eyestalk ganglia with a specific major form during the molt cycle. Effect of ecdysteroids on the levels of *CasMIH* transcript in the eyestalk ganglia has been investigated by *in vitro* incubation studies with different concentrations and ratios of ecdysteroids. In addition, the effects of ecdysteroids acting through *CasEcR* were examined by *in vivo* RNAi studies.

Molting is one of the most important physiological processes in the animals belonging to the phylum Arthropoda, and it recapitulates throughout their life cycle. Crustacean molting and the molt cycle are coordinated and controlled by the levels of hormones produced by two endocrine systems: eyestalk ganglia and Y-organs. It has been suggested that animals at the intermolt stage carry low levels of ecdysteroids due to high levels of MIH that suppresses the activity of Y-organs. The suppression of MIH on Y-organs is noted with a reduction of protein synthesis and cholesterol uptake in these organs, possibly resulting in the reduction of ecdysteroid synthesis and secretion [54,55]. However, the binding affinity of Y-organ membranes to MIH as well as the levels of its second messenger, cGMP, does change significantly during a molt cycle [17]. On the other hand, the maximum number of binding sites increases significantly at post molt (B) stage [17]. These data indicate that the inhibitory effects of MIH on the Y-organ may not result from the binding capacity or affinity of receptors. Interestingly, it is found that at the premolt stage the phosphodiesterase (PDE) in Y-organs increase and affect the levels of cAMP and cGMP in *P*. *clarkii*, thus alleviating the MIH/CHH actions [56]. However, the exact mechanism by which MIH regulates Y-organ activity through the molt cycle remains unknown.

The levels of *CasMIH* expression in the eyestalk ganglia vary and increase at early premolt stage. This result is rather different from those reported in *C*. *maenas* in which the expression levels remain constant [17]. In addition, the levels of CasMIH in the hemolymph and sinus gland appear to be higher at premolt than those of *C*. *maenas* [18]. Our results indicate that the transcription of *CasMIH* may be under the regulation of ecdysteroids. Indeed, the sequence of upstream promoter region of *CasMIH* (GenBank accession no. KJ813010, Fig 7) contains the binding sites of EcR-RXR complex and broad complex, similar to that reported in *C*. *pagurus*, *Metapenaeus ensis* [57,58] and *Charybdis feriatus* (GenBank accession no. AF092945.1) [59].

If *CasMIH* expression is under the control of ecdysteroids, the eyestalk ganglia should respond to ecdysteroids in the hemolymph and show the increase in its binding capacity to ecdysteroids at premolt stage, compared to the intermolt. In order to answer this question, the ecdysteroid contents in the eyestalk ganglia and Y-organs that were obtained from the animals at different molt stages were measured. The results show that there are indeed different levels of ecdysteroid concentrations between these tissues. Ecdysteroid levels at premolt eyestalk ganglia are higher than those at the intermolt. In contrast, the ecdysteroid levels in intermolt and premolt Y-organs are similar but higher than those from eyestalk ganglia at both molt stages. It indicates that the eyestalk ganglia are more sensitive to the hemolymphatic ecdysteroids than the Y-organs. More importantly, eyestalk ganglia show molt-stage dependent binding capacities to ecdysteroids. An elevation of ecdysteroid amounts may have occurred by increasing the number or binding affinity of its receptor. The exact type of ecdysteroids in the tissues has not been characterized. However, we suggest that PoA and/or 20-HE may be the ones present in these tissues.

Hemolymphatic ecdysteroids change during the molt cycle in both the total concentrations and the levels of a particular active ecdysteroid [34]. Ecdysteroids mimicking the levels of early premolt stage that consist of PoA:20-HE at a 3:1 ratio increase *CasMIH* expression in the intermolt eyestalk ganglia. Our results are pertinent to an earlier finding in *C*. *antennarius* that ecdysteroids stimulate MIH production [35]. Interestingly, only an ecdysteroid concentration that is similar to that of an early premolt stage stimulates the *CasMIH* expression of the eyestalk ganglia obtained from intermolt animals; however, the highest concentration (250ng/ml) suppresses its expression. The latter result appears to be contradictory to the data shown in Fig 1A where the eyestalk ganglia obtained from premolt stages (D_1/2_ and D_3-4_) have levels of *CasMIH* transcripts similar to those of early premolt. However, as shown in Fig 3, it may be due to the inherent limitation in ecdysteroid binding capacity of the intermolt eyestalk ganglia.

At premolt, the increase in the total concentration of ecdysteroids is due to the elevated levels of PoA, yielding the ratio at 3:1 between PoA and 20-HE. Such changes in total levels and PoA may indicate relative changes in *EcR/RXR* expression as well. The signal transduction of ecdysteroids requires a heterodimer complex of EcR and its binding partner RXR. Interestingly, our results show that the distribution of *CasEcR* isoforms differs by molt stages. In eyestalk ganglia, *CasEcR1* expression contributes around 70% towards total *CasEcR* expression. Since the LBP of CasEcR1 seems to be less hydrophobic than that of CasEcR2, CasEcR1 may prefer to bind to 20-HE rather than PoA. However, levels of *CasMIH* expression in the eyestalk ganglia incubated with a 1:3 ratio between PoA and 20-HE do not differ from the controls (Fig 4B). On the other hand, the incubation of ecdysteroids at a 3:1 ratio that mimicked intrinsic condition of early premolt (75ng/ml) significantly and specifically increases the levels of *CasMIH* expression, while it has no effect on the levels of *CasAK* expression. Thus, it appears that the initial elevation of ecdysteroids, particularly PoA, may be important, specifically in regulation of *CasMIH* expression.

Our aim was to examine the initial stimulatory effect of ecdysteroids on *CasMIH* expression and we carried out 3 hr incubation studies. Levels of *CasEcR* in the eyestalk ganglia do not change compared to those measured in the control group. EcR is known to be an early-ecdysteroid responsive gene; however, there may be a critical and limited window of its expression after the exposure to ecdysteroids. Indeed, upregulation of *EcR* expression in limb buds of *U*. *pugilator* occurs only at 1.5 hr incubation with 20-HE but not at 2 or 2.5 hr incubation [53]. Thus, it is implied that upregulation of *CasEcR* might have taken place earlier, which was then subsequently translated for transducing the ecdysteroid signal and *trans*-activating *CasMIH* expression as shown in Fig 4. However, further studies are required, particularly for establishing a relationship between the levels of transcription and translation of EcR in crustaceans using a time-course incubation study.

CHH neuropeptides specifically produced in crustacean eyestalk ganglia tend to accumulate in the sinus gland similar to adipokinetic hormone in corpora cardiaca in insects [60,61]. The progress of life cycle accentuates their accumulation, suggesting that the amounts of MIH/CHH translation are far greater than what is required by the animal. However, >90% reduced levels of *CasMIH* trancripts do not significantly induce molts.

Interestingly, a long-term administration of *CasMIH-dsRNA* seems to be lethal as a quarter of the animals died during the mid-premolt (D_1_ and D_2_) stage. This might have occurred as off-target effects of *CasMIH*-*dsRNA* [62–64]. However, it is also suggested that a lack of CasMIH may affect the synthesis pathway of ecdysteroidogenesis in Y-organs in terms of the levels and types of active ecdysteroids as reported in eyestalk-ablated crabs. At premolt stage, the eyestalk ablated *C*. *sapidus* produced 20-HE as the major ecdysteroid, while the intact animals generated PoA as the major form [34]. Although this aspect has not been clarified in the current study, it will be intriguing to examine in the future, if indeed multiple injections of *CasMIH-dsRNA* may replicate a similar effect that is seen with the eyestalk ablation.

The same amounts of multiple injections of *CasEcR1*-*dsRNA* reduce amounts of *CasMIH* transcripts, strongly suggesting that transcription of *CasMIH* may be under the control of ecdysteroids. However, *CasEcR1-dsRNA* injections seem to be less effective in knockdown of *CasEcR1* expression, compared to those determined with *CasMIH-dsRNA* injections. The knockdown efficiency of a target gene by RNAi depends on the levels of its expression as well as the function or property of that particular gene product [65–67]. We suggest, however that this result may be due to the low translation rate of *CasEcR* or the slow turnover of CasEcR in the eyestalk ganglia.

An upstream open reading frame as well as an internal-ribosome entry site present in the 5´untranslated region of crustacean *EcR* cDNAs [44] implicate a tight regulation in translation of crustacean *EcR* including *CasEcR*. It indicates that translation of the *CasEcR* mRNAs may require a particular physiological condition(s). In addition, the reduced levels of *CasEcR* transcripts by 67% may have a much greater impact on its protein levels. As a master transcription factor, the reduction of CasEcR levels will interfere with the transcription of its responsive genes. As alluded to earlier, the upstream promoter region of *CasMIH* gene contains the binding sites for EcR-RXR and BR-C. Therefore, reduction of *CasMIH* expression upon *CasEcR1-dsRNA* injection supports the fact that levels of *CasMIH* expression respond to ecdysteroids. However, only an injection of *CasEcR1*-*dsRNA*, not of *CasRXR-dsRNA*, reduces *CasMIH* expression by 70%. It is because EcR directly binds to ecdysteroids, while its partner RXR facilitates the liganded EcR to bind to the ecdysteroid-responsive elements.

**Fig 8:**
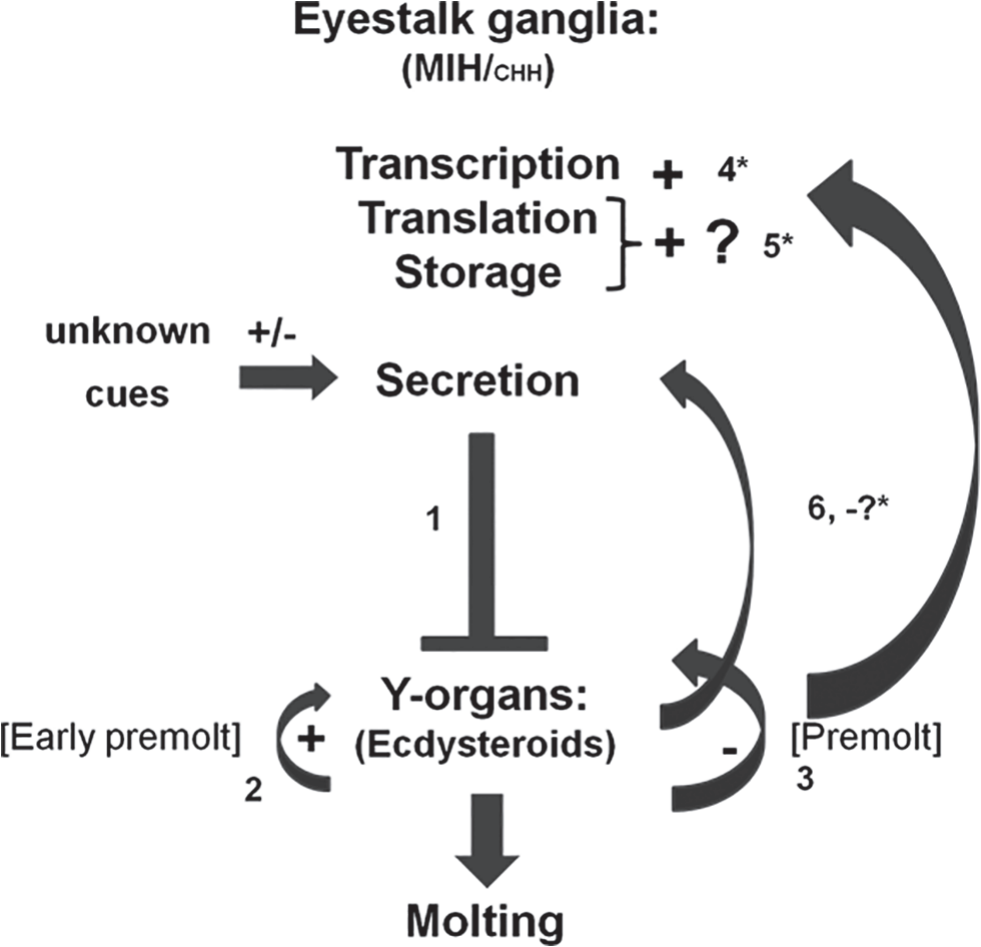
A proposed model of molt control in crustaceans. (1) Neuropeptides (MIH/CHH) secreted from the sinus gland of eyestalk ganglia suppress the ecdysteroidogenesis in Y-organs [16,17]. Autocrine feedbacks of ecdysteroids on the Y-organs may be generated from different ecdysteroid levels: (2) Ecdysteroid concentrations at early premolt stage act positively on ecdysteroidogenesis, while (3) the high levels at the premolt stage inhibit the ecdysteroidogenesis in the Y-organs as reported in *C*. *maenas*, *O*. *limosus* and *U*. *pugilator* [37–39]. This study provides supporting evidence that ecdysteroids at premolt have a stimulatory effect on eyestalk ganglia for neuropeptide production, particularly at transcriptional level (4), generating the long-loop feedback on Y-organs. Based on the endogenous changes of *CasMIH* transcripts in the eyestalk (X-organ) and protein levels in the sinus gland in this study, ecdysteroids may also have a positive effect on transcription (+, 4*), translation (?, 5*) and storage (+,5*). On the contrary, with low concentrations of MIH measured in hemolymph at ecdysis of this study (noted as-?), ecdysteroids may suppress the secretion of neuropeptides as reported in *C*. *antennarius* [35](6). Overall, this current model of molt control includes the interaction(s) between ecdysteroids and neuropeptides towards coordinating the molting process that recapitulates throughout the life cycle of decapod crustaceans. The present study contributes asterisks shown in the proposed feedback pathways, while ‘?’ requires further studies.

With the data obtained from this study including the direct effects of ecdysteroids on the *CasMIH* expression, together with the previous findings [35,36,57], a regulatory mechanism for molt control is proposed using *C*. *sapidus* as a model (Fig 8). At the early premolt, elevated levels of ecdysteroids may stimulate the *MIH* expression in eyestalk ganglia that is confirmed by an *in vitro* incubation of ecdysteroids as well as *CasEcR1-dsRNA* injections. The exact cue for MIH release has not yet been identified in crustaceans. However, the levels of hemolymphatic ecdysteroids may negatively affect the release of CasMIH as low levels of hemolymphatic CasMIH are measured at D_2_ stage, when the ecdysteroids peak, similar to an earlier report in *C*. *antennarius* [35]. The highest levels of CasMIH in the hemolymph are found at post-molt stage during which animals have the lowest levels of hemolymphatic ecdysteroids (Fig 2). Such high levels of CasMIH preceding intermolt stage would inhibit the synthesis and release ecdysteroids by the Y-organs, resulting in re-setting of the molt cycle.

This overall proposed model for molt control describes the two feedback mechanisms involving MIH in the eyestalk ganglia and ecdysteroids produced by Y-organs. Firstly, the long-loop feedback includes the neuroendocrine axis between the eyestalk ganglia and Y-organs in that MIH inhibits the activity of the Y-organs at intermolt and post molt stage. At premolt stage, elevated levels of ecdysteroids stimulate the synthesis of the MIH but suppress its release in eyestalk ganglia. In addition, it suggests that different levels of ecdysteroids may have the short-loop feedback for stimulating and inhibiting the ecdysteroidogenesis in Y-organs. Similar to other steroid hormones, the regulatory activities of ecdysteroids suggest that they are acting through EcR. However, further studies for examining these following topics are required: 1) the levels of CasMIH translation in X-organs at different molt stages in response to the varying concentrations of ecdysteroids; 2) the binding affinity of a specific EcR on each active ecdysteroid type; 3) such elicited effects by different ecdysteroids on *MIH* expression in the eyestalk; 4) the direct effects of PoA and 20-HE on Y-organs, and 5) the regulation of CasMIH secretion from the sinus gland, especially at ecdysteroids elevation and the postmolt stage.

## Materials and Methods

### Animals and sample collections

Male and female young juvenile *C*. *sapidus* (20–40 mm carapace width, CW) obtained from the Blue Crab Hatchery, Aquaculture Research Center (ARC, Institute of Marine and Environmental Technology, Baltimore, MD, USA) were reared in the same conditions as stated [44] until they reached the size of 50–90 mm CW (crab stage 13–16 [68]). The stadium of each molt stage of these animals as stated [68], is as follows: intermolt (C_4_: 12–30 days), and premolt (D_0_:,3–7 days D1: 3–7 days, D_2_: 1–3 days, and D_3-4_: 1–3 days). After the collection of hemolymph as described [44], the animals were placed on ice for 5–10 min prior to dissection. Eyestalk ganglia and Y-organs were dissected under a stereomicroscope and frozen immediately on dry-ice. The samples were stored at -80°C until further use.

### Temporal distributions of ecdysone receptor (*CasEcR*) isoforms in eyestalk ganglia at different molt stages

Eyestalks were collected from three animals at the following molt stages: intermolt: C_4_, early premolt: D_o_, mid-premolt: D_1-_D_2_, and late premolt: D_3-4_. Tissues were homogenized with 150 μl ice-cold DEPC treated PBS and 100 μl of homogenized samples were subjected to total RNA extraction, as described [44,69]. After treating with DNase I (Fermentas), the total RNAs (1.5 μg) were subjected to the 1^st^ strand cDNA synthesis using RevertAid Reverse Transcriptase (Fermentas). The cDNA samples were diluted to the final concentration at 12.5 ng total RNA equivalent/μl. Each sample cDNA (1 μl) was examined using an end-point RT-PCR assay with GoTaq Green Master Mix (Fermentas) and isoform-specific primers at the following PCR condition: initial denaturation at 94°C for 3 min; 30 cycles at 94°C for 30 s, 58°C for 30 s, and 72°C for 30 s; and a final extension at 72°C for 7 min (Table 1). The PCR products were separated on a 1.5% agarose gel and were documented using a Kodak gel-documentation system. The result as shown in Fig 5A is the representative picture of three independent assays.

**Table 1:** List of primers that were used for qPCR assays and an end-point RT-PCR assay.

Primers	Sequences (5'-3')
***CasEcR***	
qF-short (EcR1/EcR2)	CCAACAAGTCCAATGTCAGCCGGGG
qF-long (EcR1a/EcR2a)	CACTCCTAGCATCGTTCAGACT
qR1 (EcR1/EcR1a)	GGCATCCGAGTCGTCATCACTTATT
qR2 (EcR2/EcR2a)	CATGTCACTTGTATCTTCACCATCG
EcRt-qF	GCATTGTGTTTGGAAATACCTTGCC
EcRt-qR	GCCCTCAATGCATCGAGGTATATTT
***CasAK***	
qF	TTCCTCCACCCTGTCCAA
qR	GAAGCGGTCACCCTCCTTGA
***CasMIH***	
qF	TCCCTCGCTCACTCCAAATTTTC
qR	ATTGATAACTCTCGCCGCTGCTT

### *In vitro* incubations of ecdysteroids on *CasMIH* expression in eyestalk ganglia

The animals (80–90 mm CW) at intermolt were ice-chilled; the eyestalks were dissected under a dissection microscope; and, the retinas were removed carefully and rinsed with ice-cold DEPC-treated crustacean saline (pH 7.4). The eyestalks were pre-incubated in DEPC-treated crustacean saline, 0.05% M199 media (incubating media) for 30 min at room temperature for pre-conditioning to an experimental condition. The eyestalks were rinsed in the incubation media and then incubated with the incubating medium containing PoA and 20–20-HE at a 3:1 ratio (w:w). These concentrations (75, 150, and 250 ng/ml) of ecdysteroids for the incubation studies were chosen, based on the levels of intrinsic circulating ecdysteroids at D_o_, D_1_, and D_2_ stages, as reported in *C*. *sapidus* [34,44]. The eyestalk ganglia were incubated on a shaker (30 rpm) for 3 hr at 23–25°C. The incubated eyestalk ganglia were rinsed twice with DEPC-treated crustacean saline, blotted on a Kimwipe to remove the saline before being immediately frozen on dry-ice, and kept at -80°C until further analyses.

### Total ecdysteroids in tissues using ecdysteroid-radioimmunoassay (Ecd-RIA)

Eyestalk ganglia and Y-organs at various molt stages were homogenized in 100 μl ice-cold DEPC-treated PBS and dissolved in 0.1 M borate buffer (pH 8.0). The homogenized samples were assayed similarly as described [34]. The concentration of ecdysteroids was calculated using AssayZap program (BIOSOFT) and presented in pg/tissue. Protein contents in eyestalk ganglia, Y-organs, and hepatopancreas were estimated using a colorimetric assay, modified Lowry method (Pierce 660).

### MIH titers in hemolymph using RIA

#### A) Iodination

Native CasMIH was isolated and quantified as described (Chung and Webster, 2003). 300 pmol of CasMIH was iodinated by following the procedure as described [17,70]. ^125^ICasMIH was separated on a PD-10 column (GE Healthcare) and diluted in glycerol (final 50%). Then it was stored at 4°C and used within one or two months after iodination. The specific activity of ^125^ICasMIH ranged from 300 to 500Ci/mmol.

#### B) Hemolymph sample preparation

Hemolymphs were sampled from the animals at various molt stages. Hemolymph was directly withdrawn into a marine anticoagulant [71] at a ratio of 1:1 (v/v) via the arthrodial membrane between a chela and the first walking leg. These samples were extracted in isopropanol (final 60%) and centrifuged as described [72]. The supernatants were recovered in Minisorb tubes (Nunc), dried by vacuum centrifugation (Jouan), and assayed in duplicate. Anti-CasMIH was used at a 1:20,000 final concentration as before [73]. Standards of CasMIH RIAs ranged from1.2 X10^-12^ and 2.5 X 10^-11^M with ED_50_ values of 5.7 ± 0.1 ×10^-10^M (n = 3) and the detection limit of 1.2 x 10^-12^M. The RIA data were analyzed as above.

### Knockdown of *CasMIH* expression in eyestalk and molt interval using double-stranded RNA (*dsRNA*)

*CasMIH*-, *CasEcR1*-, and *CasRXR*-*dsRNA*s were generated using TranscriptAid T7 High Yield Transcription kit (Fermentas) as described [74]. Prior to the experiments, the hemolymph (100 μl) was withdrawn from the all experimental crabs (60–70 mm CW), mixed in a marine anticoagulant at a 1:1 ratio [71] and kept at -20°C for measuring ecdysteroid levels. The knockdown effect with *dsRNA*s was determined in molting event by injecting ~30 times over 60 days. Animals received 15 μg Cas*MIH-dsRNA* (in 100 μl crustacean saline) or Cas*EcR1-dsRNAs* three times a week as described [74]. The controls received the same volume of crustacean saline. In order to monitor the delivery of *dsRNA*s into the animals, the crustacean saline was spiked with a 0.01% phenol red as described [74]. At the end of the experiments, the animals were molt-staged as intermolt stage [68]. After collecting the hemolymph samples, they were then anesthetized by chilling on ice for 5–10 min and the tissue samples were collected as above.

### Expression levels of *ecdysone receptor* (*EcR*) and *molt-inhibiting hormone* (*MIH*) using qRT-PCR assays

The eyestalk ganglia at different molt stages, from incubation and *dsRNA* injection experiments, were initially homogenized in 150 μl of ice-cold DEPC-treated PBS. Total RNA extraction and cDNA synthesis were carried out as described above. The cDNA samples were diluted to the final concentration at 12.5 ng total RNA equivalent/μl. The eyestalk cDNA samples (25 ng of total RNA equivalent) were assayed in duplicate to estimate the levels of *CasMIH* and *CasEcR1* using Fast SYBR Green Master Mix (Applied Biosystems) and qRT-PCR primers (listed in Table 1) on an Applied Biosystems 7500 instrument. The expression of *arginine kinase* (*CasAK)* was determined in the same sample cDNAs as a reference gene for endpoint PCR and qRT-PCR assays. The standards of all transcripts analyzed were generated similarly as described [69,72].

### Sequencing analysis of upstream promoter region of *CasMIH gene*

Genomic DNA (gDNA) of *C*. *sapidus* was extracted from a piece of heart (~20 mg wet weight) using a Qiagen DNA extraction kit. The quality of gDNA was first examined on a gel after a partial digestion with Dral restriction enzyme. The gDNA was processed using a Universal GenomeWalk kit (Clonetech) for genomic amplification. Procedures for subcloning of PCR products were as described [44] and the sequences were analyzed using ConSite (http://consite.genereg.net/).

### Statistical analysis

All results represent mean ± 1SE (n), in which n is the number of replicates. SigmaPlot (version 12.3) was used to evaluate the statistical significance of the results. The data were subjected to the normality test using the Shapiro-Wilk test (SigmaPlot) prior to all statistical tests. The data that did not show a normal distribution were analyzed using nonparametric ANOVA (Mann-Whitney Rank Sum test). In all cases, statistical significance was accepted at *P* <0.05 and noted with letters or asterisks.

## Supporting Information

S1 Fig A time course study of *CasMIH-dsRNA* injections on the levels of *CasMIH* expression in eyestalk ganglia. The knockdown effects on *CasMIH* transcripts after 5, 7, and 10 times injection. Each cDNA sample containing 25 ng total RNA equivalent was assayed in duplicate. The expression levels are represented as copies/μg total RNA. The data are presented as mean ± SE (n). All data were subjected to a normality test using the Shapiro-Wilk test (SigmaPlot). When the data did not show the normal distribution, a nonparametric test (Kruskal-Wallis One Way Analysis of Variance on Ranks) was employed. Statistical significance was accepted at *P* < 0.05 and noted with letters.
(DOCX)
