# Supplementary figures and images for: Ecdysteroids Regulate the Levels of Molt-Inhibiting Hormone (MIH) Expression in the Blue Crab, Callinectes sapidus
Source: PLoS One. 2015 Apr 7;10(4):e0117278. doi: 10.1371/journal.pone.0117278 (PMC4388526; doi:10.1371/journal.pone.0117278)

**Techa and Chung, Suppl. Figure S1**

10
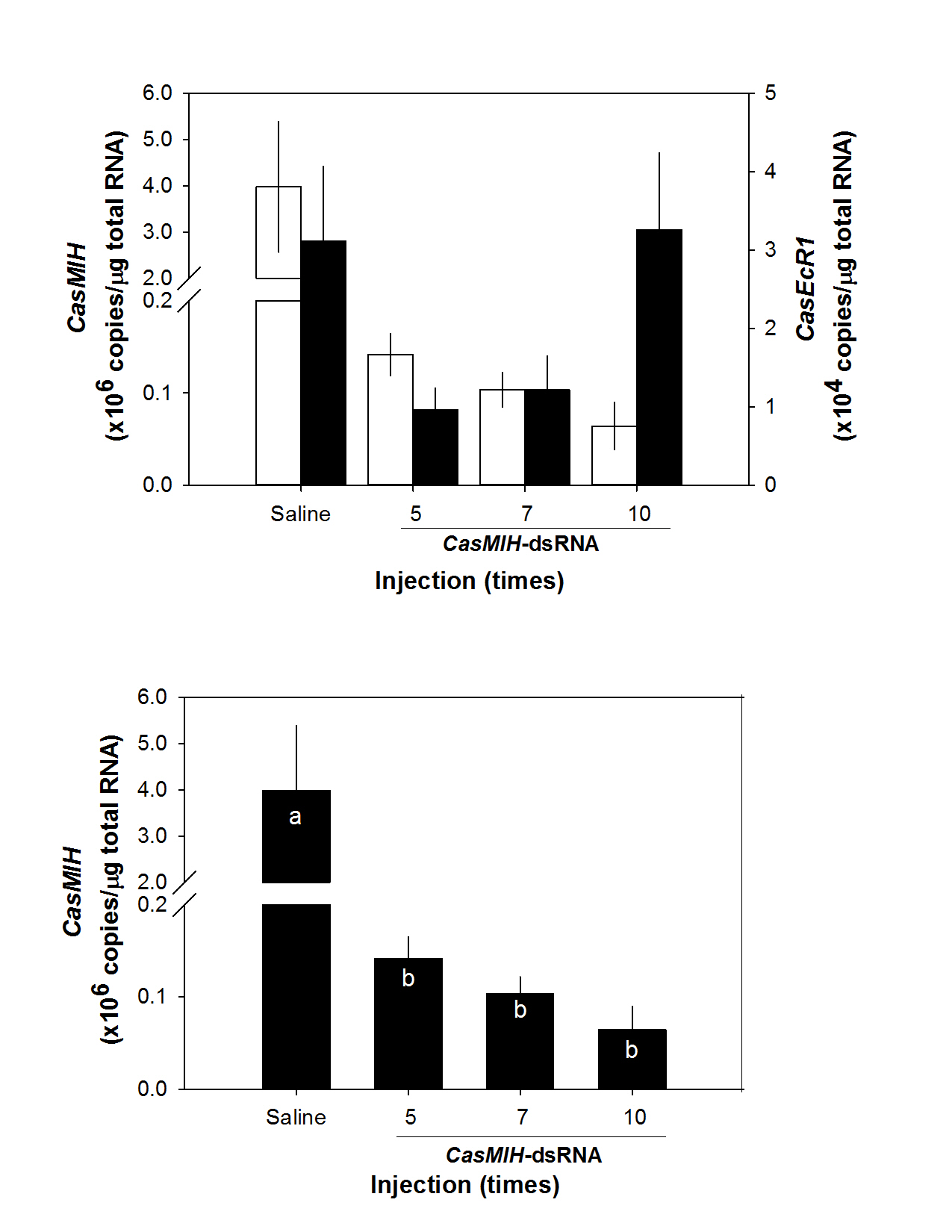

Supplement: S1 Fig — The knockdown effects on CasMIH transcripts after 5, 7, and 10 times injection. Each cDNA sample containing 25 ng total RNA equivalent was assayed in duplicate. The expression levels are represented as copies/μg total RNA. The data are presented as mean ± SE (n). All data were subjected to a normality test using the Shapiro-Wilk test (SigmaPlot). When the data did not show the normal distribution, a nonparametric test (Kruskal-Wallis One Way Analysis of Variance on Ranks) was employed. Statistical significance was accepted at P < 0.05 and noted with letters. (DOCX) [file pone.0117278.s001.docx]
